# Patients with cluster headache show signs of insomnia and sleep related stress: results from an actigraphy and self-assessed sleep study

**DOI:** 10.1186/s10194-023-01650-w

**Published:** 2023-08-18

**Authors:** Caroline Ran, Felicia Jennysdotter Olofsgård, Anna Steinberg, Christina Sjöstrand, Elisabet Waldenlind, Anna Dahlgren, Andrea Carmine Belin

**Affiliations:** 1 Centre for Cluster Headache, Department of Neuroscience, Karolinska Institutet, Stockholm, Sweden; 2 Department of Clinical Neuroscience, Karolinska Institutet, Stockholm, Sweden; 3 Department of Neurology, Karolinska University Hospital, Stockholm, Sweden; 4 Department of Neurology, Danderyd Hospital, Stockholm, Sweden

**Keywords:** Sleep, Sleep disturbance, Headache, Pain, Circadian rhythm

## Abstract

**Background:**

Cluster headache (CH) is a primary headache disorder which is characterized by circadian timing of headache attacks, usually at nighttime, in around two thirds of patients. Patients with CH often report sleep difficulties, though it is unknown whether this is a cause or a consequence of nightly headache attacks.

**Objective:**

In this case-control study we have assessed sleep quality in study participants with CH in cluster bout respectively in remission, compared to a control group of neurologically healthy individuals to investigate the potential connection between sleep and CH.

**Methods:**

Fifty study participants with CH and 42 controls were recruited for sleep assessment. Sleep was recorded using MotionWatch 8 actigraphs (CamNTech) for a period of two weeks. Study participants were instructed to wear the unit during rest and sleep and to fill out a sleep diary daily through the two-weeks period.

**Results:**

Results from actigraphy recordings and sleep diaries suggested that patients with CH spend longer time in bed than controls (CH 8.1 hours vs. Controls 7.7 hours, *p*=0.03), but do not sleep more than controls (CH 6.7 hours vs. controls 6.5 hours, *p*=0.3). In addition, CH patients reported increased sleep latency (*p*=0.003), particularly during, but not restricted to, cluster bouts. Study participants with CH further reported higher levels of stress at bedtime (*p*=0.01), and they felt less well rested than controls (*p*=0.001).

**Conclusion:**

Our analysis suggests that sleep is negatively affected in CH both in cluster bout and in remission, manifesting in symptoms consistent with insomnia such as prolonged sleep latency and increased time in bed.

**Supplementary Information:**

The online version contains supplementary material available at 10.1186/s10194-023-01650-w.

## Introduction

Cluster headache (CH) is a severe primary headache classified as a trigeminal autonomic cephalalgia [1]. Most patients with CH have bouts with daily headache attacks interspersed with symptom free periods (remission), this phenotype is labeled episodic (ECH), as compared to chronic CH (CCH) where remission periods are shorter than three months per year. A characteristic for the disease is the extreme pain experienced by CH patients during attacks [2], and also that headaches occur with patterns of diurnal rhythm (at specific timepoints during the day) for a large majority of patients [3, 4]. Multiple studies report that the most common time for attacks to appear is in the middle of the night [4–7].

Sleep and circadian rhythm are frequently discussed as potential players in CH pathophysiology. It has been generally difficult to dissect cause and consequence, as the occurrence of nightly headache attacks will disturb the patients’ sleep. Orexin A and melatonin, two hormones involved in sleep and arousal, have been detected at lower levels in patients with CH [8, 9]. Some patients also benefit from melatonin as a prophylactic treatment [10]. Activation of the hypothalamus, which regulates circadian rhythm, has been observed during CH attacks, and deep brain stimulation to the hypothalamic area has shown potential in treating intractable CCH [11–13]. Genes regulating the circadian rhythm have been proposed as candidate genes for CH [12, 14, 15]. Apart from biological evidence, clinical/epidemiological data also points to sleep disturbances among CH patients. CH patients have been suggested to suffer from insomnia to a higher extent than healthy individuals in a Norwegian cohort [6].

Sleep as well as lack of sleep have been suggested as trigger factors for CH attacks when patients are in an active bout [4, 6]. Rapid eye movement (REM) sleep dysregulation has been proposed in CH, as well as the occurrence of CH attacks specifically during REM sleep. However, other studies challenge the causality between REM sleep and nightly CH attacks [16–19].

A recent study using actigraphy and sleep diaries to assess sleep in ECH patients found impaired sleep specifically during active bouts when compared to a matched control group. Patients in bout were found to have increased sleep time, and increased time in bed. Interestingly, no significant differences were found between patients in active bout and patients in remission suggesting patients in remission might be in an intermediate state of impaired sleep [20]. We set out to investigate these findings further in our Swedish CH cohort, using a larger number of study participants. We also included patients with CCH with the aim of achieving better statistical power in the analysis, and ultimately clarifying if and how disturbed sleep is key to the phenotype. The overall aim of this study was to explore sleep in CH patients in a large Swedish patient cohort compared to controls, using actigraphy and sleep diaries. Specifically, we aimed to analyze overall sleep and specific sleep parameters in relation to phenotype (ECH/CCH), disease status (active/remission) and the occurrence of nighttime attacks to investigate if sleep differs between these conditions. Explorative analyses were designed with the aim of addressing age and sex differences and coping mechanisms.

## Methods

Study participants to the case-control study were recruited from the Swedish Cluster Headache Biobank, which has been described in detail elsewhere [4]. Following a short phone interview (Supplementary eData 1), 50 participants with a CH diagnosis verified by a neurologist and 42 participants without CH (controls) were recruited for sleep assessment, see Table 1 for information on phenotype and demographic information. Disease status (bout or remission) was defined as self-reported regular attacks (as described in the International Classification of Headache Disorders 3rd edition, ICHD-3) during the recording period, or part of the period [1]. Participants were excluded at the interview stage if they stated that they were shift workers, had a very irregular lifestyle (in regard to sleep habits and work/school schedule), or lived outside of Sweden. A subset of study participants was excluded from the analysis, two controls and five CH patients did not start the study, and one patient lost the actigraph and did not complete the diaries. Three more patients were excluded from the study, two that recorded less than five nights and one that worked night shifts, as this was an exclusion criterion for participating in the study. We did not exclude participants who failed to complete the entire period or who failed to perform one of the measurements (see Table 1 for completion rates). Furthermore, patients were not excluded because of other health problems or any drug usage that might influence our results, instead such influences were controlled for as described in the data analysis section.

**Table 1:** Detailed information on study participants

**Status**	**Controls (*n*=42)**	**Episodic cluster headache (*n*=32)**	**Chronic cluster headache (*n*=18)**
In cluster bout^a^ (%)	NA	5 (15.6)	14 (77.8)
Average attack frequency per day^b^	NA	2.2	3.1
Bout duration in months	NA	1.6	7.8
Acute treatment (%)	NA	31 (96.9)	17 (94.4)
Intermediate treatment (%)	NA	5 (15.6)	1 (5.6)
Prophylactic treatment (%)	NA	17 (53.1)	17 (94.4)
Disease duration (years)	NA	13.6	14.2
Diurnal rhythmicity of attacks (%)	NA	23 (71.9)	13 (72.2)
Patients with nightly attacks^c^	NA	3	11
Female sex (%)	19 (45.2)	13 (40.6)	11 (61.0)
Age (years)	40.5	37.1	49.4
Living with children younger than 10 years (%)	19 (45.2)	14 (43.8)	4 (22.2)
Overall healthy^d^^, e^ (%)	40 (95.2)	25 (78.1)	6 (33.3)
Take drugs that can affect sleep^d^ (%)	0 (0)	6 (18.8)	8 (44.4)
Actigraphy analysis completed (%)	39 (92.9)	26 (81.3)	15 (83.3)
Sleep diary analysis completed (%)	39 (92.9)	22 (68.8)	15 (83.3)

^a^Chronic cluster headache patients are included in the subgroup of participants in cluster bout

^b^Data on attack frequency was retrieved from previously collected data in our biobank

^c^Number of individuals having reported the occurrence of nightly attacks during the recording period retrieved from actigraphy data and/or diaries

^d^Ranked based on answers from self-reported questions about general health and living situation, questions 6-11, Supplementary eData 1

^e^For detailed information, see eTable 1

### Objective measure of sleep

MotionWatch 8 actigraphs (CamNTech) were used to perform sleep recordings for two weeks. The MotionWatch 8 actigraph has been validated against polysomnography (PSG) with reliable results for certain sleep parameters (time in bed and wake after sleep onset), overall sleep assessment (83% overall agreement with PSG), and no systematic over- or underestimation of specific sleep parameters [21]. Participants received the actigraph units by post. Actigraphs were set to record in MotionWatch Mode 1 with an epoch of 30 seconds. Subjects were instructed to wear the actigraph unit as much as possible, but at least during sleep and rest. If the unit was removed during daytime, subjects were asked to wear it during a short active period before going to bed in the evening. The actigraph had an event button which was used by the subject to indicate when he or she was ready to sleep (as in turn the lights off) and when he or she woke up. Study participants with CH were also asked to press the button in the event of an attack occurring during nighttime sleep. All sleep recordings were performed during Swedish wintertime, specifically during the months of November 2020 to March 2021. The output variables time in bed, sleep length, sleep latency and sleep efficiency were analyzed.

### Subjective measure of sleep

During the study period participants filled out a modified version of the Karolinska Sleep Diary (Supplementary eData 2) after waking up, or as soon as possible, to avoid recollection bias. The diaries were filled out in a web-survey tool provided by Karolinska Institutet; KI survey, or in paper format if requested. The output variables were time in bed, sleep length, sleep latency, general sleep score (How did you sleep?), being rested, difficulty falling asleep and waking up, early wake-up, disturbed sleep, worry/stress, sleepiness, and last, Sleep Quality Index (SQI), which was calculated by using the mean of four sleep parameters “’ease falling asleep’, ‘sleep quality’, ‘calm sleep’, and ‘slept throughout’” according to a method developed by Keklund and Åkerstedt [22]. The SQI has previously been shown to adequately summarize subjective sleep parameters both in a laboratory setting using polysomnography with controlled sleep conditions and when investigating natural sleep cycles during normal working days [22, 23]. Being based on only four parameters, the SQI is a useful tool to study subjective sleep quality, but does not necessarily corelate with objective sleep quality and should be considered in such a light.

### Data analysis and statistics

Actigraphy data files and sleep diaries were blinded before analysis, although identifiers were removed, the data sometimes revealed if the subject was a CH patient or not (reports of occurrence of CH attacks). Actigraphy recordings were scored in the MotionWare 1.2.1 software provided with the actigraph units. Sleep diaries were used to verify the actigraphy recordings and to correct the data when the markers for sleep-time and wake-up were missing.

Individual means for each subject were calculated for all actigraphy and sleep diary variables 1) for the total period of recordings, and 2) for weekdays and weekends, before performing comparative group analysis.

The primary analysis tested for differences between cases and controls 1) on the whole cohort, 2) with respect to CCH and ECH vs. controls and 3) active bout and remission vs. controls. There was a significant overlap between the groups; CCH and active bout as well as for ECH and remission. Explorative analysis included a) subgroup analysis to investigate age and sex differences, b) analysis to verify if reported general health, lifestyle, drug usage, and occurrence of nighttime attacks affects the results, c) analysis of weekends and weekdays separately, and d) detailed analysis of bedtimes in the patient group.

To examine differences between CH and controls and/or the different subgroups, Students’ t-test was used for analysis of normally distributed data, and Mann-Whitney-Wilcoxon test was used for non-normal data. Normal distribution of data was assessed with Shapiros-Wilk test. For numerical data, such as age, linear regression analysis was used. All data analysis was performed in R studio 4.1.2 [24]. Correction for multiple testing was carried out for comparison and implies a significance level of α=0.0125 for actigraphy analysis and self-reported sleep data, and α=0.005 for subjective sleep assessment.

## Results

Fifty CH patients and 42 controls were recruited to the study. Age and sex as well as the proportion of participants who lived with young children was similar in the patient and the control groups. In the CH group there was a higher number of CCH patients (*n*=18) vs ECH patients (*n*=32) than what is seen in a random CH sample, which was due to the recruitment of CCH patients specifically to ensure a large enough sample size for sub-group comparisons. As CCH is more common in females, this also affected the ratio of males to females (1:1) with a higher-than-expected number of females, (the ratio is usually around 2-3:1 in CH cohorts), age was also more elevated in the CCH group. Five of the ECH patients were in active bout, and 27 were in remission. The attack frequency was slightly higher in CCH patients (3.1 attacks per day vs. 2.2 in ECH), as was the occurrence of nightly attacks which was reported by 3 out of 5 ECH patients in bout and 11 out of 15 CCH patients. The use of prophylactic treatment was more common in CCH patients (94.4% vs. 53.1% in ECH), while the use of intermediate treatment was more common in ECH (15.6% vs. 5.6% in CCH). Furthermore, there was a higher proportion of other diseases in the CH group as compared to the control group (38% vs. 4.7%), of those, 31.6% had diagnoses related to brain and/ or psychiatric diagnoses (eTable 1). Specifically, other health problems and usage of drugs that can affect sleep was more common in CCH patients (66.7% and 44.4% respectively) as compared to ECH patients (21.9% and 21.9%) (eTable 1). More CH patients than controls did not complete the study (20% vs. 7% and 26% vs 7% respectively for actigraphy and sleep diaries). 40 patients and 39 controls were included in the actigraphy analysis, 37 patients and 39 controls were included in the sleep diary analysis (Table 1). The average number of recorded nights was similar between the two groups (13.6 in patients and 14 in controls).

### Objective sleep measurements

**Fig. 1:**
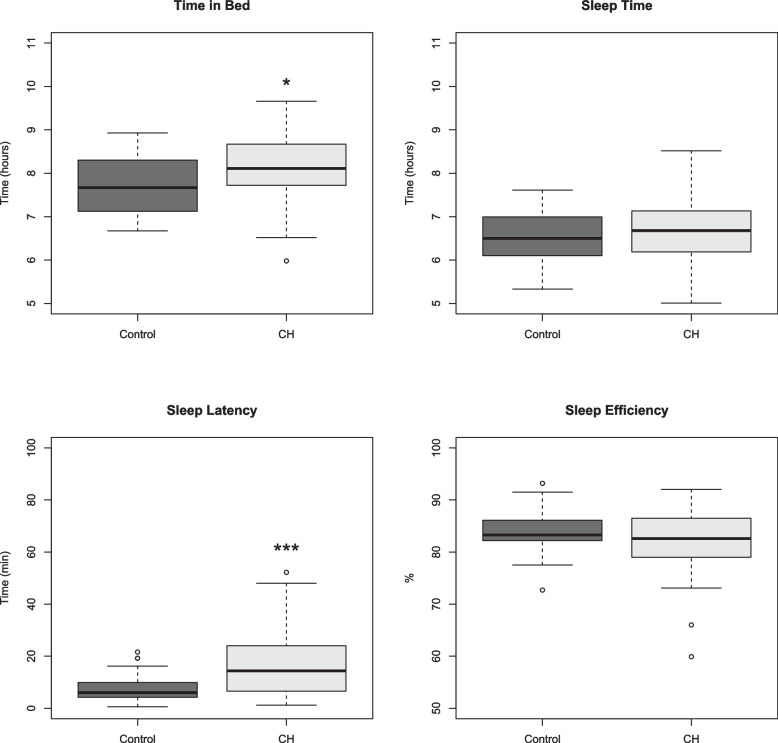
Actigraphy sleep analysis in cluster headache patients and controls. CH: Cluster Headache. Top left panel: Time spent in bed, *p*-value=0.03, Top right panel: Sleep time, *p*-value=0.3, Bottom left panel: Sleep latency, *p*-value <0.001, Bottom right panel: Sleep efficiency, *p*-value=0.2. * *p*-value<0.05, ** *p*-value significant after testing for multiple comparisons, *** *p*-value<0.001

Group analysis comparing mean hours of sleep per night as measured by actigraphy did not show any significant difference between CH patients (6.7 hours) and controls (6.5 hours), *p*=0.33, Fig. 1. Although both patients and controls overestimated their sleep in the sleep diaries (Table 2, eFigure 1). Several additional parameters from the actigraph units were analyzed to assess sleep in patients with CH (Fig. 1). Patients were found to spend longer time in bed than controls (8.1 hours vs. 7.7 hours, *p*=0.03). The sleep latency, which is a measurement of the time it takes for an individual to fall asleep, was increased in patients (17.4 minutes vs. 7.8 minutes in controls, *p*<0.001) (Fig. 1). Sleep efficiency was measured by actigraphy and did not differ statistically between the groups, although lower values were noted in CH patients (81.9%) as compared to controls (84.1%) (Fig. 1). Sleep diary data were consistent with actigraph recordings (Table 2, eFigure 1).

**Table 2:** Subjective ratings of sleep, sleepiness, and sleep related stress

**Sleep parameter**	**C**	**CH**	*p*-value	**ECH**	*p*-value	**CCH**	*p*-value	**Bout**	*p*-value	**Rem**	***p*-value**
Time in bed (hours)	7.8	8.2	0.004	8.2	0.04	8.3	0.01	8.2	0.05	8.3	0.02
Sleep time (hours)	7.1	7.3	0.4	7.3	0.3	7.1	0.9	7.0	0.7	7.4	0.1
Sleep latency (minutes)^c^	14.8	26.1	0.003	20.5	0.08	34.2	<0.001	34.6	<0.001	20.3	0.09
**Sleep scores (scale 1-5) **^**a**^											
General sleep score	3.8	3.3	<0.001	3.4	0.006	3.1	0.007	3.2	0.008	3.4	0.006
Well-rested after waking up	3.1	2.7	0.001	2.9	0.1	2.3	0.001	2.4	0.002	2.9	0.06
Difficulty falling asleep^c^	4.3	3.9	0.002	4.1	0.1	3.5	<0.001	3.5	<0.001	4.1	0.06
Waking up too early^c^	4.5	4.1	0.006	4.2	0.02	3.9	0.04	3.9	0.01	4.2	0.04
Disturbed sleep^c^	4.1	3.6	0.007	3.8	0.1	3.4	0.004	3.6	0.02	3.7	0.04
Worried/stressed at bedtime^c^	4.5	4.2	0.01	4.3	0.2	3.9	0.003	4.1	0.06	4.3	0.03
Difficulty in waking up^c^	3.1	2.9	0.2	2.9	0.4	2.9	0.2	2.9	0.1	2.9	0.5
SQI^c,d^	4.2	3.7	<0.001	3.9	0.003	3.5	<0.001	3.5	<0.001	3.8	0.001
**Sleepiness scores (scale 1-9) **^**b**^											
Sleepiness at bedtime	7.0	6.9	0.7	6.9	0.6	6.9	0.8	6.8	0.6	6.9	0.8
Sleepiness at waking up	5.8	6.0	0.6	5.8	0.8	6.2	0.3	6.2	0.3	5.8	0.8

*C* Control, *CH* Cluster Headache, *ECH* Episodic CH, *CCH* Chronic CH, *Bout* CH patients in active bout, *Rem* CH patients in remission

^a^lower numbers equals to having a worse sleep score

^b^1 being “extremely alert” and 9 being “very sleepy/having difficulties to stay awake”

^c^Mann-Whitney-Wilcoxon test for non-normally distributed data

^d^method described by Kecklund and Åkerstedt [22]

*p*-value: *p*-value when compared to controls, differences between groups were analyzed using student’s t-test or Mann-Whitney-Wilcoxon test

**Fig. 2:**
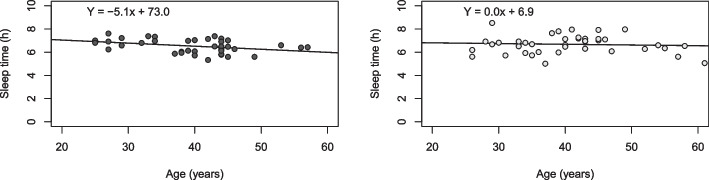
Correlation between sleep time and age in cluster headache patients and controls. Left panel: controls (dark grey dots) *p*-value from linear regression analysis=0.02, Right panel: Cluster Headache (CH) patients (light grey dots) *p*-value from linear regression analysis=0.6

In a secondary analysis, the material was divided into different strata to verify how different factors influenced sleep measurements. Sleep time was found not to decrease at older age in the CH group as it tended to do in the control group (*p*=0.02), (Fig. 2, eTable 2). Sex affected sleep in patients, with longer sleep time measured in females (6.9 hours) compared to males (6.4 hours), *p*=0.04, this association was not observed in controls (6.6 hours in females vs. 6.4 hours in male controls, *p*=0.3), (eTable 2). Living with young children (<10 years), having other health issues, or taking drugs that might affect sleep did not impact mean hours of sleep in neither patients with CH nor in controls (eTable 2).

As an additional control statistical comparisons for the actigraph measurements were repeated excluding 14 individuals who had CH attacks at night during the recording period, leaving 27 individuals with CH in the patient group. This analysis gave similar results to our analysis comprising all patients: showing no difference in sleep time (CH 6.8 hours vs. controls 6.5 hours, *p*=0.1), increased time in bed (CH 8.2 hours vs. controls 7.7 hours, *p*=0.02), increased sleep latency (CH 15.6 minutes vs. controls 7.8 minutes, *p*=0.001) and no change in sleep efficiency (CH 82.5% vs. controls 84.1%, *p*=0.5).

### Subgroup objective sleep analysis

**Fig. 3:**
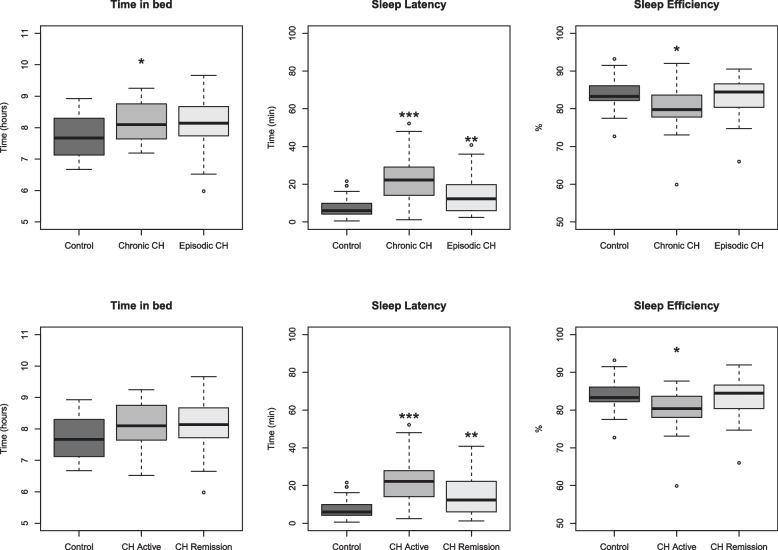
Actigraphy analysis in subgroups of cluster headache patients and controls. CH: Cluster Headache, CH Active: Cluster Headache patients in a cluster bout, Remission CH: Cluster Headache patients in remission. All comparisons were made to controls: * *p*-value<0.05, ** *p*-value significant after testing for multiple comparisons, *** *p*-value<0.001

CCH patients were analyzed in comparison to controls, showing that the total sleep time was not different in this patient group. Time in bed was longer in CCH (8.2 hours vs. 7.7 in controls, *p*=0.04), (Fig. 3). Sleep latency was also found to be increased in CCH patients (22.8 minutes vs. 7.8 minutes in controls, *p*<0.001), (Fig. 3), these findings were validated by sleep diary analysis (Table 2). Moreover, sleep efficiency was decreased in CCH patients (79.8% vs. 84.1% in controls, *p*=0.02), (Fig. 3). CCH patients did not differ from ECH for any of these four sleep measurements (data not shown), and ECH patients differed from controls only in sleep latency (ECH 14.4 minutes vs. 7.7 minutes in controls, *p*=0.004).

To investigate whether or not the sleep differences observed in CH patients were occurring exclusively when patients experience active CH bouts, CH patients who had active CH (episodic or chronic) during the duration of the study were grouped and compared to CH patients in remission and to controls. Patients in cluster bout did not differ from patients in remission on any of these sleep measurements; sleep time, time in bed, sleep latency and sleep efficiency (data not shown). When compared to controls, sleep time was not affected in active CH patients. Time in bed for active CH patients was not significantly different from controls (*p*=0.1), (Fig. 3), while sleep latency was increased in active CH patients: 22 minutes vs. 7.8 minutes in controls, *p*<0.001, and in remission (15 minutes, *p*=0.004), and sleep efficiency decreased in active patients (79.8%, *p*=0.02), (Fig. 3). When analyzing these parameters in sleep diary data, both time in bed and sleep latency were different in active bout and time in bed was increased in remission, both as compared to controls (eFigure 2).

### Subjective sleep analysis

Thirty-seven CH patients and 39 controls were included in the sleep diary analysis. Study participants assessed different parameters linked to sleep and quality of sleep each day at wake up (Supplementary eData 2). Analysis showed that CH patients scored worse in almost all self-assessed sleep quality parameters (Table 2). When asked about the general quality of their sleep on a scale from 1 to 5 (1 being the worst), CH patients had an average score of 3.3 compared to 3.8 in the control group (*p*<0.001). When asked how well-rested they felt after sleep, CH patients scored significantly lower than controls (2.7 vs 3.1, *p*=0.001). Patients also had more disturbed sleep (CH: 3.6 vs controls: 4.1, *p*=0.007) and greater difficulty falling asleep (CH: 3.9 vs controls: 4.3, *p*=0.002), (scale 1 to 5 with 1 being “least rested”/“very disturbed”/“great difficulty”). Furthermore, CH patients were more likely to wake up too early (CH: 4.1 vs controls: 4.5, *p*=0.006, scale 1 to 5 with 1 waking up “much too early”) and feel worried or stressed at bedtime (CH: 4.2 vs controls: 4.5, *p*=0.01, scale 1 to 5 with 1 being “very worried/stressed”). Moreover, CH patients had a significantly worse SQI index than controls (CH: 3.7 vs controls: 4.2, *p*<0.001, (scale 1 to 5 with 1 being the worst sleep quality).

There was no difference between CH patients and controls when it came to sleepiness when going to bed (CH: 6.9 vs controls: 7.0, *p*=0.7, and sleepiness when waking up (CH: 6.0 vs controls: 5.8, *p*=0.6), (scales 1 to 9 with 1 being “extremely alert”). Nor did we see any difference in difficulty in waking up (CH: 2.9 vs controls: 3.1, *p*=0.2, on a scale 1 to 5 with 1 being “extremely difficult”). Applying the assigned thresholds for multiple testing caused a subset of significant results to be lost.

### Subgroup subjective sleep analysis

When comparing the different subgroups such as patients in remission, patients in a cluster bout, and patients with CCH/ECH, to controls all subgroups had worse general sleep score than controls and a worse SQI score than controls (Table 2). In line with results from the general CH cohort, individuals with CCH, in a cluster bout or in remission had more disturbed sleep, were more prone to wake up too early, and were more worried/stressed at bedtime than controls (non-significant trend in active patients *p*=0.06) (Table 2). Patients with CCH or in active bout had more difficulty falling asleep and felt worse rested, as compared to controls (Table 2). ECH patients were more prone to wake up too early compared to controls (Table 2). No other parameters were significantly different between the four subgroups and controls.

### Weekdays vs. weekend sleep analysis

**Fig. 4:**
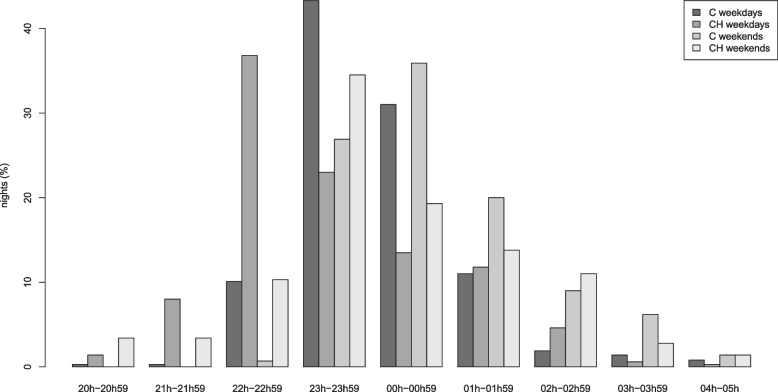
Reported bedtime in cluster headache patients and controls. Self-reported bedtime plotted as % of total number of nights separated in two categories: weekdays and weekends. 100% represents 365 weekdays in controls, 348 weekdays in cluster headache (CH) patients and 145 weekend days in both controls and CH patients

In a next step, weekends were separated from regular workdays in order to discover potential coping mechanisms, i.e., catching up on sleep over the weekend to compensate for lack of sleep or tiredness. Both the patient and the control group had longer time in bed on weekends (+54 minutes in controls, *p*<0.001 vs. +42 minutes in patients, *p*=0.001) as well as longer sleep time (+42 minutes in controls, *p*<0.001 vs. +36 minutes in patients, *p*=0.007) (eTable 3). Sleep latency was not affected on weekends in either group. Sleep diary data showed a similar pattern, except for patients with CH who estimated their sleep latency was shorter on weekends with 21.9 minutes as compared to 28.0 minutes on weekdays (*p*=0.05) (eTable 3). Self-reported bedtime was plotted in one-hour intervals, revealing that CH patients went to bed earlier than controls, both on weekdays and weekends (Fig. 4). In the early hours of the evening (20:00-22:00), CH patients were clearly overrepresented. CH patients went to bed on average at 23:16 on weekdays, which was 41 minutes earlier than the control group (*p*<0.001). In the weekends, patients with CH had an average bedtime of 00:05, as compared to controls who went to bed 36 minutes later, at 00:41 (*p*<0.001).

## Discussion

In this study we have performed a thorough investigation of sleep quality and patterns in patients with CH, using actigraphy in combination with sleep diaries. Actigraphy and sleep diary estimates were quite similar and showed that even though CH patients and controls slept on average the same amount per night, sleep was affected in CH with increased sleep latency, longer time in bed, and overall experiencing more disturbed sleep. These results confirm findings from previous sleep studies showing increased sleep latency using PSG, and increased time in bed with actigraphy [19, 20, 25]. We could not replicate previously reported lower sleep efficiency for the CH whole group of patients, possibly because of the large number of episodic patients in remission in our study, or the superior measurements obtained with PSG. Sleep time, which was significantly longer in Danish patients in bout was not different from controls in our cohort. The difference between studies may be due to the composition of the cohort, as our analysis included chronic patients. When applying correction for multiple testing to our data, not all sleep parameters remained significant. Specifically increased time spent in bed in CH patients only showed a trend for association after correcting for multiple tests (the same was observed in subgroup analysis). Taking into account the similar findings of the studies discussed above, the overall results point to the same direction, suggesting sleep is affected in CH patients. However, replication of our findings in other CH cohorts is warranted.

Sleep diary analysis revealed that patients with CH had increased stress and anxiety at bedtime, experienced more difficulties falling asleep, more often woke up too early, had more disturbed sleep and worse quality of sleep as compared to controls. Patients did not feel more tired than controls either at bedtime, nor at wakeup, and they did not have difficulties waking up, but were contrarily feeling less well rested than controls. Applying correction for multiple testing slightly impacted our results, being stressed at bedtime did not remain significant, while other parameters remained significant or showed a strong trend for association. Similarly, in the subgroup analyses some *p*-values do not hold correction for multiple testing, but SQI remained significantly different in all analysis. Overall, the data clearly show that CH patients experience their sleep to be worse than do controls. The objective measures of sleep showed that CH patients took longer time to fall asleep and spent longer time in bed. Together this might indicate a sleep behavior related to worry about getting enough sleep, which with the concurrent sleep related stress and anxiety may be symptoms of insomnia [26]. The ratings of worse sleep quality in CH patients were not shown in the objective measures of sleep. Actigraphy does not measure the different sleep stages, but previous studies using PSG have demonstrated increased REM latency in CH patients [19, 25]. Future studies should examine if the subjective ratings of worse sleep in CH patients may reflect differences in sleep architecture (e.g., less deep sleep and/or micro arousals). The finding that CH patients went to bed earlier than controls may be translated into a behavior compensating for sleeping difficulties as discussed above, in particular in light of the finding that CH patients were not more tired at bedtime. On the other hand, there was no evidence of increased sleep time on the weekends in the patient group, which may be expected in individuals with impaired sleep. An alternative interpretation of the shift in sleep-times towards earlier hours observed in the patient group would be a difference in circadian rhythm. The possibility of CH patients having a shift or a dysregulation in the molecular clock resulting in a different circadian behavior is not fully elucidated and investigations of chronotype in CH patients are so far inconclusive [3].

Sleep time was analyzed in different subgroups of patients and controls to investigate how personal factors may influence our results. Age was found to be associated with decreased sleep time in controls, but not in CH patients, a finding that may indicate that patients with CH require more sleep as they grow older to compensate for the strain that the disease imposes [27]. As the circadian rhythm changes with age, such dysregulation could also result in age-related phenotypes. Further, female patients with CH were found to have increased sleep time compared to male CH patients. This result is also consistent with previous findings showing that female patients are more severely affected by CH than male patients [28].

Analyzing CH patients with different phenotypes showed that CCH patients had a more severe sleep impairment than ECH patients. In addition to the sleep parameters discussed above, CCH patients displayed a sleep efficiency below 80%, which is considered below the normal variation of sleep efficiency [29]. As CCH patients often have a higher disease burden, a more pronounced sleep disturbance was not surprising in this group of patients. Moreover, CCH patients on average scored worse on the self-reported sleep-assessment items in the sleep diary. The prevalence of nightly attacks was somewhat higher in the group of chronic patients than in episodic patients in bout and this may also influence our results as patients with more nightly attacks have interrupted sleep. In our analysis 11.5% in the ECH subgroup presented with nightly attacks during the recording, as compared to 73.3% of the CCH patients and therefore these groups need to be compared with caution. Having nightly respectively daily attacks can impact a patient’s life in different ways, as nightly attacks are less visible to the surrounding society but can also have a detrimental impact on the patient’s quality of sleep and thereby an effect on the general well-being. Sleep and CH have always been discussed in terms of cause and consequence, as it seems obvious that a pain disorder manifesting during sleep results in disturbed sleep. It is known that the CH diagnosis can transform into the other subtype (ECH/CCH) during the life of a patient, and it is therefore highly relevant to extend the division of patients beyond the traditional classification of subtypes. Here, several subgroup analyses were performed to try to understand whether sleep impairments were due to patients waking up in the night as a result of nightly CH attacks or if sleep is impaired also in patients who sleep through their nights. When stratifying patients in an active cluster bout, we found very similar results to the CCH subgroup analysis. A limitation of our study was the difficulty of identifying ECH patients in a cluster bout, and therefore it should be noted that the subgroups of “CCH” and “active CH” overlap to a large extent, and by consequence also the results. Interestingly, these analyses showed that ECH patients and CH patients in remission (also overlapping) showed signs of sleep impairment similar to those observed in CCH/active patients. Even though sleep efficiency was not different from controls in these subgroups, sleep latency was prolonged and significantly different from controls. In contrast to our data showing sleep differences in patients in remission, Lund et al. previously did not find differences in any sleep parameters between controls and patients in remission [20]. This difference in results may be due to the larger material analyzed in our study, in combination with the small amplitude of the changes in sleep. Lund also analyzed differences between ECH in bout and in remission and found no differences. Interestingly, on inspection of the data, it seems as if that the patients in remission presents intermediate values, and opens the possibility of a linear relationship between the severity of CH and the severity of sleep problems. These data are in line with ours, and are confirmed by trends in the PSG reports [19, 25]. Although the severity of sleep problems may depend on the current status of a patients’ CH (active/remission), all our analyses are consistent with sleep impairments occurring in CH patients regardless of their headache burden. An internet-based survey study on European patients has previously shown that patients in remission also experience stress and worry for future attacks and avoid potential triggers for attacks [30]. Analyzing only patients who did not have nighttime CH attacks during the recording period provided another piece of evidence supporting our results of sleep impairment being a general problem in CH patients. These results were in accordance with results from the Danish study using actigraphy, which did not see any differences in patients with nocturnal CH attacks [20].

Our study was not designed to dissect the reasons behind sleep impairments in CH, but we may speculate that there are several contributing factors. Psychological factors such as worrying of having an attack during the night or worsening of symptoms due to lack of sleep may be reflected in patients in active bout having a harder time falling asleep compared to patients in remission. Lack of sleep has been reported to be a trigger factor for CH attacks [28], and there are reasons to continue the investigation of underlying biological factors, such as sleep hormone regulation, circadian rhythm, and hypothalamic involvement. Melatonin is sometimes used in the treatment of CH, and has shown a positive effect on attack frequency [31]. More thorough evaluation of the use of melatonin and other sleep interventions in the treatment of CH is warranted both with regards to headache symptoms, and sleep difficulties. Sleep and pain are two interlinked physiological processes, and lack of sleep is known to exacerbate pain. One study found that individuals without headaches had a 40% increased risk of developing headaches if they had previously reported symptoms of insomnia [32]. Another study previously reported that sleep problems, along with drinking caffeine and presence of other pain was associated with developing new headache episodes [33]. This is further complicated due to effects of caffeine on sleep, and findings that CH patients have a higher energy drink consumption than controls [34]. It is worth noting that we did not account for caffeine and/or energy drink consumption in our study. The directionality of the association between sleep and pain has long been discussed, but recent studies point towards a stronger effect of sleep on pain than of pain on sleep from a temporal perspective [35–38].

PSG is considered the gold standard for sleep measurements within the field due to its accuracy and ability to measure sleep stages which our actigraphy watches are unable to do. Unlike actigraphy, PSG can additionally measure breathing disturbances and eye and leg movement and is primarily used in the clinical setting for diagnosing sleep-disorders [39] However, actigraphy offers the advantage of being performed in the home environment of the study participant, and during a longer time period than PSG, which ensures more stable measurements and cancels out the first night effect commonly seen in sleep-laboratory measurements also in good sleepers. It has previously been suggested by Lund et al. [25] that actigraphy might present an advantage specifically in analyzing sleep in CH patients, as the stress associated with sleeping in a new environment might affect the occurrence of attacks. They report that less patients than expected had nightly attacks during polysomnography recordings (roughly 45%), as compared to 59% of ECH patients experiencing nighttime attacks when recorded in their home with actigraphy [19, 20, 25]. This hypothesis can be confirmed in our study with 73.3% of the CCH patients, and 66.7% ECH patients reporting attacks during the recordings. For the purpose of our study, which did not include investigating sleep-disordered breathing, actigraphy offered a non-invasive method to investigate sleep over an extended period of time. There are several limitations to the use of actigraphy, as mentioned above, actigraphy does not provide reliable readout of sleep-stages. Also, actigraphy is known to be better at detecting sleep than wakefulness resulting in unprecise readings of e.g. sleep time, sleep latency and sleep efficiency [21]. Specifically, actigraphy has been demonstrated to have low specificity to correctly assess periods of wakefulness during sleep [40]. Last, as actigraphy is performed in the participants home, this introduces an incertitude of how the recordings were performed, and is accompanied by a lower completion rate and occurrence of missing data which can be avoided in sleep-laboratory recordings. One of the strengths of our study is the combining of two measurements of sleep, actigraphy and sleep diaries. This provided the advantage of understanding deviations in the data, as it became possible to compare sleep disturbances and data anomalies to self-reported situations. In our study, differences between patients and controls were more pronounced when analyzed with sleep diary data than actigraphy, particularly in the analysis of sleep latency. These differences may be due to actigraphy being prone to underestimate sleep latency. It can also be a result of patients having anxiety which is known to translate to sleep misperception, for example, an impression of not being able to fall asleep [41]. Sleep diary reports have been suggested to sometimes be biased towards overestimation in good sleepers, and underestimation in poor sleepers, but this potential limitation is overcome here by our choice of multiple measurements. Last, subjective assessment of sleep through sleep diaries is an important part of assessing insomnia and are highly relevant to the patient group, as sleep difficulties are a common complaint in CH.

## Conclusion

In this study we have shown that CH patients display changes in several sleep parameters compared to controls. Patients with CH spend longer time in bed than controls and have increased sleep latency. In combination with self-reported sleep, where increased sleep related stress, difficulties falling asleep and poorer sleep were observed, our findings are suggestive of CH patients suffering from symptoms that may develop into insomnia problems. Symptoms were worse in patients in cluster bout, but persist also in remission, and are unrelated to having night-time CH attacks.

### Supplementary Information

**Additional file 1: eTable 1.** List of self-reported health issues and use of medication in the cohort.** eTable 2.** Sleep time measured by actigraphy in cluster headache patients and controls.** eTable 3.** Weekdays vs. weekend sleep analysis in cluster headache patients and controls.** eFigure 1.** Sleep diary analysis in cluster headache patients and controls.** eFigure 2.** Sleep diary analysis in subgroups of cluster headache patients and controls.** eData 1.** Participant recruitment interview.** eData 2.** Modified version of the Karolinska Sleep Diary.

## Data Availability

Anonymized raw data not published within this article will be made available after reasonable request to the corresponding author, if complying with the General Data Protection Regulation (GDPR) and the Karolinska Institutet data transfer agreement.

## Abbreviations

CH: Cluster headache
ECH: Episodic cluster headache
CCH: Chronic cluster headache
REM: Rapid eye movement
PSG: Polysomnography
SQI: Sleep quality index
GWAS: Genome wide association study
