# Supplementary material for: Patients with cluster headache show signs of insomnia and sleep related stress: results from an actigraphy and self-assessed sleep study
Source: J Headache Pain. 2023 Aug 18;24(1):114. doi: 10.1186/s10194-023-01650-w (PMC10439595; doi:10.1186/s10194-023-01650-w)
Supplement: Supplementary file 1 — Additional file 1: eTable 1. List of self-reported health issues and use of medication in the cohort. eTable 2. Sleep time measured by actigraphy in cluster headache patients and controls. eTable 3. Weekdays vs. weekend sleep analysis in cluster headache patients and controls. eFigure 1. Sleep diary analysis in cluster headache patients and controls. eFigure 2. Sleep diary analysis in subgroups of cluster headache patients and controls. eData 1. Participant recruitment interview. eData 2. Modified version of the Karolinska Sleep Diary. [file 10194_2023_1650_MOESM1_ESM.pdf]

Manuscript:

Patients with cluster headache show signs of insomnia and sleep related stress: results from an actigraphy and self-assessed sleep study

Supplementary Data

eTable 1: List of self-reported health issues and use of medication in the cohort 2
eTable 2: Sleep time measured by actigraphy in cluster headache patients and controls 3
eTable 3: Weekdays vs. weekend sleep analysis in cluster headache patients and controls...... 4

eFigure 1: Sleep diary analysis in cluster headache patients and controls 5
eFigure 2: Sleep diary analysis in subgroups of cluster headache patients and controls 6

eData 1: Participant recruitment interview.. 7
eData 2: Modified version of the Karolinska Sleep Diary 8

eTable 1: List of self-reported health issues and use of medication in the cohort

	**Controls (n=42)**	**Episodic cluster headache (n=32)**	**Chronic cluster headache (n=18)**
Other health issues (n)	4.8% (2)	21.9% (7)	66.7% (12)
Reported health issues (n)	Diabetes (1)<br>Back pain (1)	Asthma (1), Attention deficit disorder (1), Blood and bowel disease (1), Crohn’s disease (1), Diabetes (1)<br>Migraine (1), Thyroidism (1), Psoriasis (1)	Allergies (1), Asthma (2), Bipolar disorder (1), Crohn’s disease (1), Endometriosis (1), Fibromyalgia (1), High blood pressure (2), High cholesterol (1), Herniated disc (1), Lung cancer (1), Lymphoma (1), Migraine (2), Myalgic Encephalomyelitis (1), Paroxysmal hemicrania (1), Polycystic ovary syndrome (1)
Use drugs that affect sleep (n)	0% (0)	21.9% (7)	44.4% (8)
Used drugs (active substance)	NA	Cabergoline (1), Citanopran (1), Dextroamphetamine (1), Pascoflair (passiflora incarnata) (1), Sertraline (1), Melatonin (2)	Melatonin (5) and<br>Blood pressure medication (1), Budesonide (1), Lamotrigine (1), Propiomazine (3), Sertraline (2), Zolpidem (1), Zopiclone (1)

NA: Not applicable

eTable 2: Sleep time measured by actigraphy in cluster headache patients and controls

**Subgroup**	**Average sleep time in controls (hours)**	**p-value**	**Average sleep time in cluster headache (hours)**	**p-value**
Sex (Female/Male)	6.6/6.4	0.3	6.9/6.4	0.04
Age	NA	0.02	NA	0.6
Children <10 years (No/Yes)	6.9/6.4	0.08	6.8/6.5	0.2
Healthy (No/Yes)	6.6/6.5	0.6	6.7/6.7	0.9
Take drugs that might affect sleep (No/Yes)	NA	NA	6.8/6.4	0.2

NA: Not applicable, differences between groups were analyzed with t-test except for age that was analyzed with linear regression.

eTable 3: Weekdays vs. weekend sleep analysis in cluster headache patients and controls

		Controls			Cluster headache		
**Data type**	**Sleep variable**	**Week -days**	**Week -ends**	**p-value**	**Week-days**	**Week-ends**	**p-value**
Actigraphy	Average sleep time (hours)	6.3	7.0	<0.001	6.5	7.1	0.007
Actigraphy	Time in bed (hours)	7.5	8.4	<0.001	7.9	8.6	0.001
Actigraphy	Sleep latency (minutes)	7.8	7.2	0.3	18.6	16.2	0.1
Sleep diary	Average sleep time (hours)	6.9	7.7	<0.001	7.0	7.8	<0.001
Sleep diary	Time in bed (hours)	7.5	8.4	<0.001	8.0	8.9	<0.001
Sleep diary	Sleep latency (minutes)	15.9	12.6	0.06	28.0	21.9	0.05

Sleep time and time in bed were analyzed with student's t-test, sleep latency was analyzed with Mann-Whitney-Wilcoxon test.

eFigure 1: Sleep diary analysis in cluster headache patients and controls

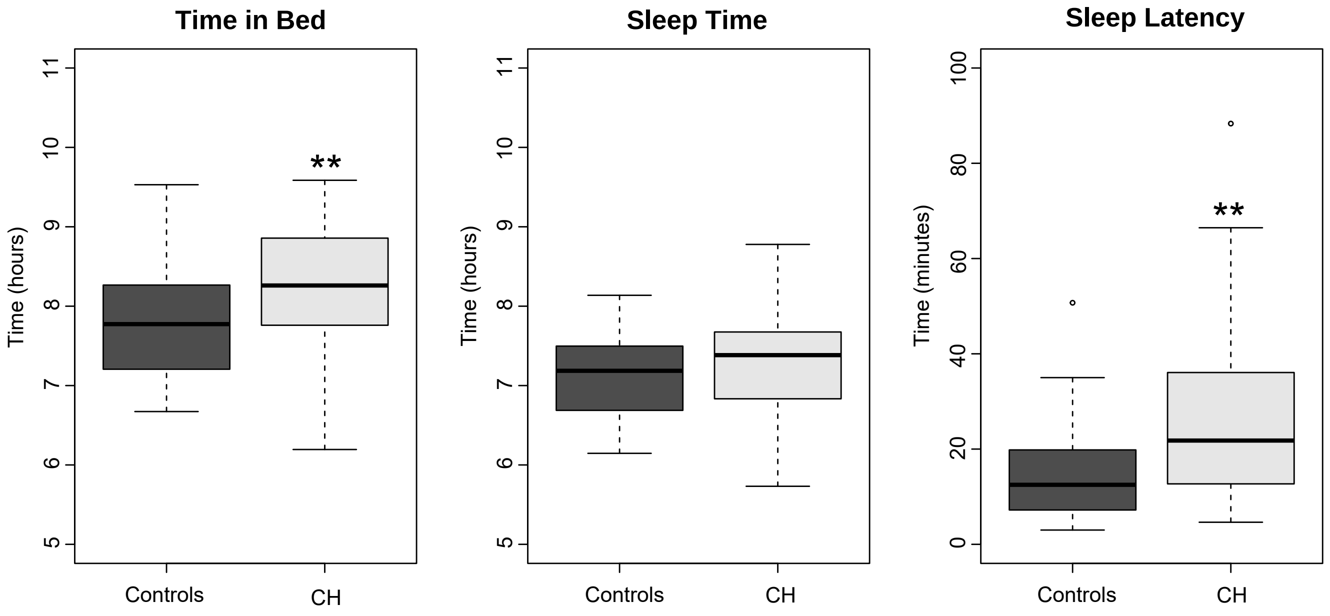

*CH: Cluster Headache, *: p-value<0.05 between cluster headache and controls, **: p-value significant after multiple comparisons, ***: p-value<0.001*

eFigure 2: Sleep diary analysis in subgroups of cluster headache patients and controls

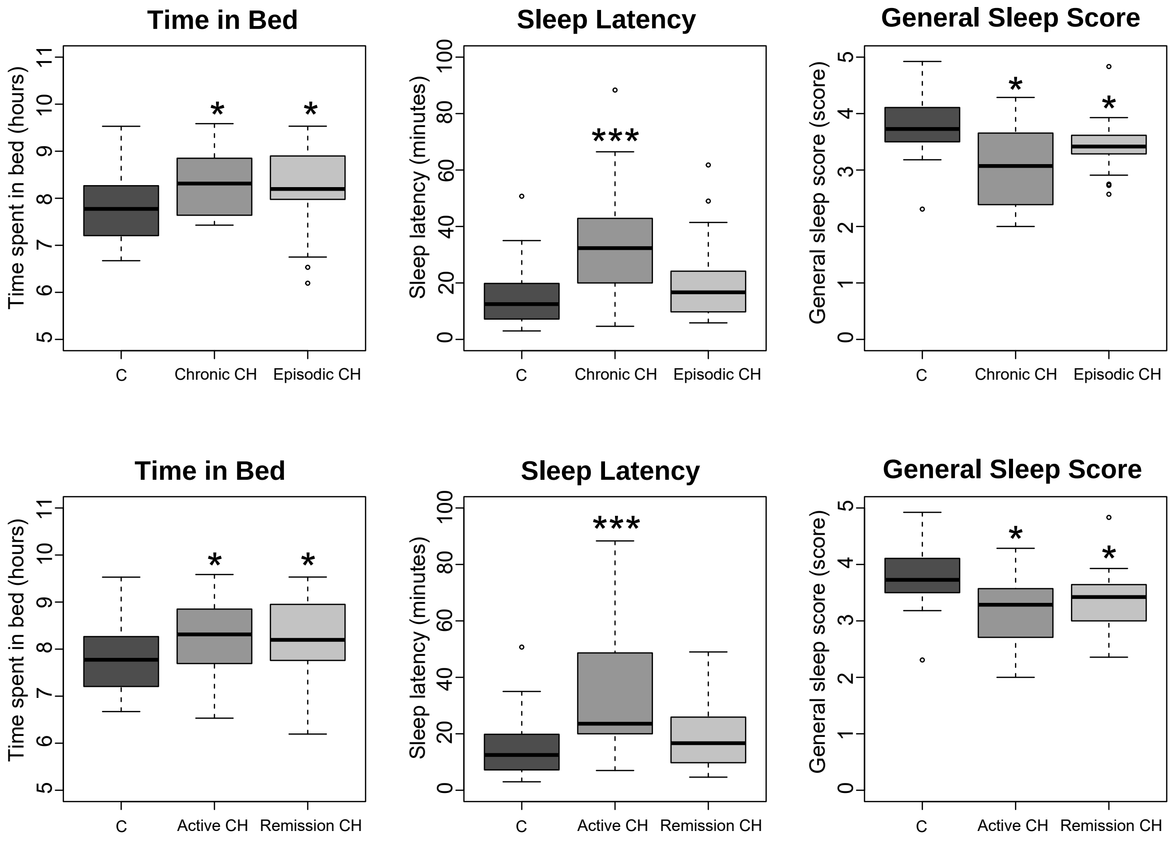

*Boxplots showing the differences between controls and cluster headache patients for three sleep parameters exported from sleep diary data. Top row, chronic cluster headache patients (Chronic CH) or episodic cluster headache patients (Episodic CH) compared to controls. Bottom row cluster headache patients in active bout (Active CH) or cluster headache patients who are in remission (Remission CH) compared to controls. *: p-value<0.05 between cluster headache patients and controls, **: p-value significant after multiple comparisons, ***: p-value<0.001*

eData 1: Participant recruitment interview

The interviewer first explains the purpose and the procedure of the study

1) Do you want to participate in the sleep study?

Questions about the cluster headaches

2) Do you have an active cluster headache period now?
3) Do you have episodic or chronic cluster headache?
4) Do your cluster headache attacks appear at specific timepoints during the day?
   a. If yes, do they appear at night?
5) Do you use any medications for your cluster headache at this time?
   a. If yes, which prophylactic drugs?
   b. If yes, which acute drugs?

Questions about general health and living situation (used as control parameters and to eliminate confounders in the data analysis)

6) Are you right or lefthanded *(information used for actigraph settings)*?
7) Are you healthy except for your cluster headaches?
8) Do you use any medications that could affect your sleep at the moment?
   For example, pain medication, sleeping pills, or medication for neurological and neuropsychiatric disorders?
9) Do you live alone or with other people?
   If living with other people:
   a. Who do you share your home with (family, children, friends etc.)?
   b. How old are the children?
10) What do you do for a living? *(shift workers are excluded)*
11) Do you work/study from home because of the current Covid-19 pandemic?

Finally the interviewer explains in more detail how the study will proceed to eligible candidates.

eData 2: Modified version of the Karolinska Sleep Diary

1) Day of measurement (the day you woke up) (YYYY-MM-DD)

2) What time did you go to bed (put the lights out) (HH:MM)

3) How long time did it take for you to fall asleep?

4) How did you feel when you went to bed?

- ☐ Extremely alert
- ☐ Very alert
- ☐ Alert
- ☐ Quite alert
- ☐ Not alert nor tired
- ☐ First signs of sleepiness
- ☐ Sleepy, but having no trouble staying awake
- ☐ Sleepy, making an effort to stay awake
- ☐ Vey sleepy and having difficulties to stay awake

5) Was it difficult for you to fall asleep?
(Mark your answer below on a 5-step scale from very to not at all)

- ☐ Very
- ☐
- ☐ Rather
- ☐
- ☐ Not at all

6) Did you feel stressed when you went to bed?

- ☐ Very
- ☐ Rather
- ☐ Somewhat
- ☐ A little bit
- ☐ Not at all

7) I woke up at (HH:MM)

8) I got out of bed at (HH:MM)

9) How did you feel when you woke up?

- ☐ Extremely alert
- ☐ Very alert
- ☐ Alert
- ☐ Quite alert
- ☐ Not alert nor tired
- ☐ Signs of sleepiness
- ☐ Sleepy, but having no trouble staying awake
- ☐ Sleepy, making an effort to stay awake
- ☐ Vey sleepy and having difficulties to stay awake

10) Did you wake up too early without being able to go back to sleep?
(Mark your answer below on the 5-step)

- ☐ A lot too early
- ☐
- ☐ Rather too early
- ☐
- ☐ No

11) Do you feel well rested?
(Mark your answer below on the 5-step scale)

- ☐ Not at all
- ☐
- ☐ Rather
- ☐
- ☐ Completely

12) Was it easy to get out of bed?
(Mark your answer below on the 5-step scale)

- ☐ Very hard
- ☐
- ☐ Not hard nor easy
- ☐
- ☐ Very easy

13) How did you sleep?
(Mark your answer below on the 5-step scale)

- ☐ Very badly
- ☐ Rather badly
- ☐ Not badly nor well
- ☐ Quite well
- ☐ Very well

14) Did you have restless sleep?
(Mark your answer below on the 5-step scale)

- ☐ Very
- ☐
- ☐ Rather
- ☐
- ☐ Not at all

15) Did you have any cluster headaches during the night?

16) If yes, please elaborate (how many, duration, did you take any medications etc)

17) Did anything else happen during your sleep?

THANK YOU!
